# Decoupling the Contributions of ZnO and Silica in the Characterization of Industrially-Mixed Filled Rubbers by Combining Small Angle Neutron and X-Ray Scattering

**DOI:** 10.3390/polym12030502

**Published:** 2020-02-25

**Authors:** Mariapaola Staropoli, Dominik Gerstner, Aurel Radulescu, Michael Sztucki, Benoit Duez, Stephan Westermann, Damien Lenoble, Wim Pyckhout-Hintzen

**Affiliations:** 1 Luxembourg Institute of Science and Technology, L-4422 Belvaux, Luxembourg; stephan.westermann@list.lu (S.W.); damien.lenoble@list.lu (D.L.); 2 GOODYEAR S.A., L-7750 Colmar-Berg, Luxembourg; dominik_gerstner@goodyear.com (D.G.); benoit_duez@goodyear.com (B.D.); 3 Forschungszentrum Jülich/MLZ Garching, D-85748 Garching, Germany; a.radulescu@fz-juelich.de; 4 European Synchroton Radiation Facility, F-38000 Grenoble, France; sztucki@esrf.fr; 5 Forschungszentrum Jülich, D-52425 Jülich, Germany; w.pyckhout@fz-juelich.de

**Keywords:** SANS, SAXS, silica, zinc oxide, partial structure factors, small angle scattering

## Abstract

Scattering techniques with neutrons and X-rays are powerful methods for the investigation of the hierarchical structure of reinforcing fillers in rubber matrices. However, when using only X-ray scattering, the independent determination of the filler response itself sometimes remains an issue because of a strong parasitic contribution of the ZnO catalyst and activator in the vulcanization process. Microscopic characterization of filler-rubber mixtures even with only catalytic amounts of ZnO is, therefore, inevitably complex. Here, we present a study of silica aggregates dispersed in an SBR rubber in the presence of the catalyst and show that accurate partial structure factors of both components can be determined separately from the combination of the two scattering probes, neutrons, and X-rays. A unique separation of the silica filler scattering function devoid of parasitic catalyst scattering becomes possible. From the combined analysis, the catalyst contribution is determined as well and results to be prominent in the correction scheme. The experimental nano-structure of the ZnO after the mixing process as the by-product of the scattering decomposition was found also to be affected by the presence or absence of silica in the rubber mixture, correlated with the shear forces in the mixing and milling processes during sample preparation. The presented method is well suited for studies of novel dual filler systems.

## 1. Introduction

Nanofiller particles added to elastomers are known to form hierarchical structures varying from clusters of the order of a few nm up to agglomerates in the range of several μm. As a consequence of this, the resulting composite materials show strongly improved mechanical properties, which lead to important applications as, e.g., most prominently in tires [1,2,3,4]. The complex arrangement of the nanoparticles in the mixtures is thereby at the basis of the associated reinforcing mechanisms. The understanding of the correlation between the filler structure and the ultimate macroscopic properties of the filled rubbers widely employs Small Angle Scattering (SAS) methods [5,6,7,8,9,10,11]. 

These techniques exploit the different sensitivity of the respective radiation probe for the components in the mixture, allowing the identification of the structural distribution of the different species. Whereas for Small Angle X-ray Scattering (SAXS) the difference in the electron density between typical soft and hard constituents determines the contrast, in the case of Small Angle Neutron Scattering (SANS) different contrasts depending on nuclear properties rather than on electronic ones may exist. In the ideal case, these contrasts can be tuned in order to elaborate the scattering of specific components only [12,13,14]. For multicomponent systems, the combination of both SAXS and SANS can be therefore used to highlight the contribution of different components to the total scattering function, due to the different but complementary scattering contrasts obtained on the same sample. The underlying manuscript will deal with an application of these two probes to identify the ZnO structure for the first time in such industrial silica-containing composites, allowing its subtraction from the structural contribution of the reinforcing fillers. 

ZnO is typically used as an activator in the vulcanization process and is known to improve the tire abrasion resistance and heat transfer at braking as well as to reduce the rubber shrinking during the molding process [15,16,17].

For conventional ZnO nanoparticles with a radius between 20 and 90 nm [18], however, the scattering contribution will occur in the same scattering vector range as the silica filler aggregates. This interference impairs, therefore, a direct evaluation of the scattering response of the reinforcing fillers, which is the only component determining the performance of the rubber. The problem of the interference and also additional inhomogeneities have been discussed in the literature [5,6,14,19,20,21,22]. Due to the difficulty in disentangling the contribution from the catalyst and the filler particles, the ZnO contribution was sometimes neglected or a simple weighted subtraction of a ZnO-containing background was applied as data correction. Recently, a more accurate correction was reported [22], based on different scattering techniques. The weighted subtraction assumed a priori an identical contribution of ZnO in filled and non-filled compounds. In addition, more recently, the use of contrast variation in such mixtures was discussed. With anomalous small angle X-ray scattering (ASAXS) at the Zn edge, which can be realized only at synchrotron sources varying the energy of the X-ray beam, a certain variation of the scattering length of the ZnO might be obtained, making it only partially contributing to the total intensity [19]. Alternatively, but for the case of neutron scattering another contrast variation procedure, in which the nuclear polarization of hydrogen atoms was modified, was applied for such compounds. However, the latter is technically difficult and is not compatible with standard neutron instrumentation [23]. In the underlying work using a combination of scattering techniques, we aim to extract the form and structure factors belonging to the silica filler complex in the presence of the full cure package and simultaneously of the necessary presence of ZnO.

Dealing with these problems is an important aspect for an enhanced understanding and the development of structure-property relationships. For the extraction of the single contributions to the total scattering function, we make use of a polymer-based approach in analogy to the Singular Value Decomposition (SVD) method widely applied to multi-component ‘green’ nanocomposites in the presence of solvents [14,24] but which relies on several samples with different labeling and contrasts. For our purpose, the unfilled rubber will be treated as a three-component mixture formed by the processing oil, matrix polymer, and ZnO [25], all leading to different scattering contrasts. The same approach is then extended to the case of silica-filled compounds in order to separate the contribution of silica and ZnO. This approximation is reasonable as these components have the strongest scattering contribution due to not only their concentration in the mixture. As we show, the approach relies on the combination of X-ray and neutron scattering intensities on identical samples and on the different contrast factors involved in the two methods to decouple the partial contributions to the total intensity. The ZnO correction is required, and its contribution appears in all samples analyzed.

## 2. Materials and Methods 

### 2.1. Samples and Preparation

The SBR used in this work has high styrene (40%) and medium vinyl content (24%). The random copolymer is characterized by a glass transition temperature of −34.5 °C and is pre-mixed with 37.5 phr (parts in weight per 100 parts of rubber) of an aromatic distillate oil (TDAE). The rubber was mixed with amorphous precipitated silica (1165 MP, Rhodia/Solvay Group, Aubervilliers, France) of a specific surface of 165.8 m^2^/g (N2 BET). Several samples with different silica volume fractions were prepared in a standard way [21] using an internal mixer of the Banbury type, followed by a two-roll milling process. Rubber and fillers were mixed in the non-productive stage using bis-3-triethoxysilylpropyldisulfide (Si266) as a coupling agent. In this stage n-(1,3)-dimethylbutyl-n-phenyl-p-phenylenediamine was used as an antioxidant while N -cyclohexyl-2-benzothiazolesulfeneamide and diphenylguanidine, respectively, as first and secondary accelerators. During the productive stage, the curing package was introduced, including ZnO with a specific surface of 5 m^2^/g used as the catalyst. Samples with different silica mass fractions: 0 (A), 30 (B), 60 (C) phr, and an unfilled sample free of ZnO (E) were studied in this work. The sample list and the silica filling degree are specified, respectively, in Table 1 and Table 2. After the milling process, the slabs were cured by compression-molding vulcanization at 170 °C for 10 min in a hydraulic press, and square sheet samples of thickness ~0.7mm were obtained. 

### 2.2. Small Angle X-Ray Scattering (SAXS)

SAXS experiments were performed at ID02 [26], ESRF, (Grenoble, France) at detector distances 31, 8, and 2 m and at a wavelength 1 Å. The usable scattering vector *q*, defined as $q=\frac{4\pi }{\lambda }\sin\frac{\theta }{2}$, extended from 5 × 10^−4^ < *q* < 0.4 Å^−1^. *θ* is the scattering angle. The q-range differs along *x* and *y* direction due to the asymmetric beam stop. 2D data were binned to 1920 × 1920 channels with 0.09 × 0.09 mm^2^ width. They were corrected for empty beam, dark current, and radially averaged (where possible) using standard procedures of the beamline. Absolute scattering intensities in [cm^−1^] units were obtained by calibration with the scattering of water at room temperature. A background, which was assigned to the left wing of the amorphous halo, was subtracted linearly as only data up to roughly *q* = 0.1 Å^−1^ were used. For reference sake, sample E was re-measured and calibrated independently vs. a glassy carbon standard at the University of Erlangen. Intensities were found to match in excellent agreement.

### 2.3. Small Angle Neutron Scattering (SANS)

SANS experiments were conducted at the KWS-2 diffractometer of MLZ, (Garching, Germany) using three detector distances of respectively 20, 8, and 2 m at a wavelength of 10 Å. The scattering vector range spanned between 1 × 10^−3^ < q < 0.4 Å^−1^. 2D-detector data were obtained in 142 × 142 channels of 8 × 8 mm^2^ width. The wavelength distribution $\frac{\Delta \lambda }{\lambda }$ was 10%. For the absolute scaling to [cm^−1^] the incoherent scattering level of a secondary standard Plexiglass was used. The two-dimensional data in 142 × 142 channels were corrected pixelwise for empty beam scattering, detector sensitivity, and background noise using B_4_C as beam blocker and subsequently radially averaged. In the case of anisotropic scattering patterns, sectors with total opening angles of 10° along the main axes of the anisotropic patterns were applied to the scattering patterns. The incoherent background arising from the hydrogenous polymer fraction and the other additives was determined from the high *q*-range and was found to be in good agreement with the estimated incoherent scattering. Likewise, as for SAXS, sample E was re-measured and put to an absolute scale at the KWS-3 diffractometer of MLZ using the direct beam method. Moreover, here, the calibrated intensities of KWS-2 and KWS-3 matched ideally.

## 3. Results

The scattering model approach used in this work is developed for the non-filled samples with and without ZnO and further extended to the case of filled systems. Due to the strong scattering of the ZnO catalyst, this component cannot be neglected in the analysis despite its irrelevance in the reinforcing mechanism and mechanical performance of the rubber. It leads, therefore, to treatments that necessarily involve its structure. Although the matrix is ideally expected to contribute as a simple background to the scattering of filled rubbers, it is known that inhomogeneities due to the crosslinks possibly lead to an additional contribution [13,14]. This contribution is normally observed in the lowest *q* region as a characteristic power law. The pure vulcanized SBR/oil sample has often been modeled then by means of a classical Ornstein-Zernike approach in analogy to the case of a semi-dilute polymer solution or swollen gel assuming that the processing oil acts as a contrast agent for the meshes of the network [25]. In the presence of the curing activator, on the other hand, at low *q* ZnO nanocrystals or domains could become visible due to their strong and different contrast with the embedding matrix and at high *q* they might exhibit Bragg peaks. The overall dimensions of the ZnO crystals could be estimated in principle with an additional contribution taken into account by a Debye-Bueche law. In the literature, the simple incoherent superposition of Ornstein-Zernike and Debye-Bueche laws was applied on a regular basis with limited success [25,27,28,29]. However, the estimation of the ZnO contribution to the total scattering could be more complex due to its interaction with other components of the mixture as sulfur and accelerators, which can be absorbed on the ZnO particles surface. These interactions could lead to a different average contrast between the ZnO and the matrix and possibly to a non-negligible deviation from the incoherent sum of two contrast-weighted partial structure factors. 

The coherent scattering intensity of a general three-component mixture I(q) is theoretically written as

(1) $$I\left(q\right)=\overset{n=3}{\underset{i,j=1}{\sum }}\rho_{i}\rho_{j}S_{ij}\left(q\right)$$

where the pre-factors of the partial structure factors $S_{ij}\left(q\right)$ are their scattering length densities $\rho_{i},\rho_{j}$ (here abbreviated as SLD). The total scattering function is then defined as a linear combination of all partial structure factors resulting from all the intra ($S_{ii}$) and inter-component ($S_{ij}$) correlation functions. The approach is first applied to the determination of different contributions in non-filled samples, which can be approximated to two- and three-components systems. In the following section, we discuss first the case of the unfilled rubber and generalize the same concept then to the reinforced case.

### 3.1. The Unfilled Case

In the following section, the polymer-based approach will be applied to the description of the scattering function of an unfilled rubber. Sample E consists of the rubber mixture but was vulcanized without ZnO. The SBR and oil constitute then a classical two-component system. On the contrary, Sample A is already a three-component but two-phase mixture containing SBR polymer, processing extender oil, and ZnO. The other curing agents can be neglected in scattering evaluations. Taking sample A as the working example in further treatment and making use of the incompressibility condition, one obtains for the intensity [25]:

(2) $$I\left(q\right)={\left(\rho_{pol}-\rho_{oil}\right)}^{2}S_{pol}+{\left(\rho_{ZnO}-\rho_{oil}\right)}^{2}S_{ZnO}+2\left(\rho_{pol}-\rho_{oil}\right)\left(\rho_{ZnO}-\rho_{oil}\right)S_{pol-ZnO}$$

Where $\rho_{pol}$, $\rho_{oil}$ and $\rho_{ZnO}$ are the SLD for the matrix, oil, and ZnO, respectively. Here, the oil is considered as the background component (or solvent in the polymer-based approach). $S_{pol},\;S_{ZnO}$ are the partial structure factors respectively for the polymer and the ZnO while $S_{pol-ZnO}$ takes into account the interaction between these two components. The q-dependence of the structure factors was omitted for readability. The intensity from a background of oil does not appear explicitly therefore in the q-dependent scattering functions. It can be easily seen that this general three-component treatment for sample A can be simplified to obtain the case of sample E. If no ZnO is present in the compound, then the second and third term of the equation vanish, and the intensity for the resulting two-component system is written in the usual well-known form as $I\left(q\right)={\left(\rho_{pol}-\rho_{oil}\right)}^{2}S_{pol}$ For sample E, the incompressibility condition leads to $\emptyset_{pol}+\emptyset_{oil}=1$. ∅ corresponds to the volume fraction of the components. The scattering contrast remains, therefore, a delicate fitting parameter. For sample A, within the approximation that only the main components contribute to the scattering function, the following condition is valid: $\emptyset_{pol}+\emptyset_{oil}+\emptyset_{zn}=1$. The small amount of activator allows neglecting interactions between neighboring ZnO nanocrystals. Therefore, the scattering contribution of the activator could ideally be described in terms of a particle form factor. Furthermore, the low concentration, as well as the non-specific interaction of the inorganic ZnO particles with the oil or SBR, are very good reasons to neglect also the cross-term $S_{pol-ZnO}$ in the scattering equation of the three-component system [12,25,29,30] which is then reduced to:

(3) $$I\left(q\right)={\left(\rho_{pol}-\rho_{oil}\right)}^{2}S_{pol}+{\left(\rho_{ZnO}-\rho_{oil}\right)}^{2}S_{ZnO})$$

This approximation applies to sample A containing less than 1% of ZnO (2.5phr). However, the contrast factors in this solid cross-linked or vulcanized mixture differ from those of the system in which ZnO would be considered as one component in an effective matrix of SBR and oil due to interaction with the curing package. The subtraction of $S_{ZnO}$ from the scattering function is, therefore, not straightforward and requires an estimation of the contrast factors involved. Moreover, the size of the particular ZnO component is a pre-requisite for adequate correction. Different scenarios could determine the effect of the ZnO on the scattering function of the filled rubber. In the case of large ZnO particles, the Guinier regime of this component is expected at too small scattering vectors q. The only contribution would, therefore, be its surface scattering with its approximately observed characteristic q^−4^ slope in addition to the Ornstein-Zernike behavior expected for the rubbery matrix. This particular q^−4^ at a low scattering vector occurs in the case of smooth particles that have a well-defined flat and strong interface with the surrounding matrix. In the case of rough or fractal surfaces, however, the detected power law would differ from −4. The power law exponent X depends on the surface fractal dimension $D_{s}$ by X = ($6-D_{s}$). On the other hand, if the mixing and milling process results in better dispersed, relatively small ZnO particles, their form factor would interfere with the scattering contribution resulting from the fillers and corrections that require the estimation of contrast factors become necessary. Unlike a SVD method based on a number of contrast parameters that exceeds the number of unknown partial structure factors [12,13,14,23,27,29,30], in our particular case, it is impossible to induce additional contrast factors into a cross-linked rubber without a previous labeling of components by, e.g., deuteration and inevitably leading to intrinsically different samples [25]. For this reason, the decomposition of the measured signal intensity has been performed using a linear combination of experimental data obtained by X-ray and neutron scattering measurements applied to the same sample. Due to the different contrast factors in the two experiments, the partial structure factors for fillers and ZnO can be extracted for each q in analogy to the SVD approach. The resulting set of two linear equations with two unknowns, i.e., the partial structure factors can be solved exactly. On the other hand, the merging of SANS and SAXS data into one evaluation may be affected by the different q-resolution of the two methods. Especially in the case of SANS, resolution and polydispersity effects are fully correlated. In the lowest q range, this does not affect the data. Resolution- and non-resolution corrected data differ only in the order of 2%–3% and do not influence the results. As such, the determination of the scattering functions strongly depends on the quality of the absolute calibrations as well as on the correct evaluation of the SLD parameters for all the components taken into account within this approximation. Therefore, absolute care in the absolute normalization of the intensities is important.

In our presented approach, the partial form factors result from the exact solution of a system of two linear equations. For sample A the expression of $S_{pol}$ and $S_{ZnO}$ is given by:

(4) $$S_{pol}=\frac{{\left(\rho_{ZnO}-\rho_{oil}\right)}_{X}^{2}I_{AN}-{\left(\rho_{ZnO}-\rho_{oil}\right)}_{N\;}^{2}I_{AX}}{{\left(\rho_{pol}-\rho_{oil}\right)}_{N}^{2}{\left(\rho_{Zn}O-\rho_{oil}\right)}_{X}^{2}-{\left(\rho_{pol}-\rho_{oil}\right)}_{X}^{2}{\left(\rho_{ZnO}-\rho_{oil}\right)}_{N\;}^{2}}$$

(5) $$S_{ZnO}=\frac{{\left(\rho_{pol}-\rho_{oil}\right)}_{N}^{2}I_{AX}-{\left(\rho_{pol}-\rho_{oil}\right)}_{X}^{2}I_{AN}}{{\left(\rho_{pol}-\rho_{oil}\right)}_{N}^{2}{\left(\rho_{ZnO}-\rho_{oil}\right)}_{X}^{2}-{\left(\rho_{pol}-\rho_{oil}\right)}_{X}^{2}{\left(\rho_{ZnO}-\rho_{oil}\right)}_{N\;}^{2}}$$

The indices N and X stand for neutron and X-ray, respectively. $I_{\mathrm{AN}}$ and $I_{\mathrm{AX}}$ correspond to the experimentally measured absolute intensities of sample A in both N and X case. They are corrected for instrumental backgrounds of any source (see Experimental). It is obvious that the contrasts as weighting factors are of extreme importance in further evaluation. Therefore, SLDs of the components, as well as the consistency of absolute calibration of intensities, have to be verified with the greatest care. We remind that the structure factor is defined as S(q)=ϕ V P(q) with V the volume of the scattering entity and P(q)  its q-dependent form factor [25]. The weighed subtractions in Equations (7) and (8) were obtained in the common SANS *q-* range after a cubic spline was fitted to the SAXS data.

### 3.2. Contrast Considerations

As discussed above, deriving accurate partial structure factors of single components that give rise to the experimental scattering intensity requires accurate estimations of the contrasts of the particular components in the mixtures. Contrast is generally defined as the square of the difference in scattering length densities (SLD) between two scattering entities. Whereas for neutrons the scattering length b  is a nuclear property, for X-rays the equivalent parameter is a function of the electronic number of the component. The SLD is then the sum of the scattering lengths per volume and is defined for neutrons and X-ray respectively as: $\;SLDN=\frac{\sum_{i=1}^{n}b_{i}}{V_{molecule}},\;SLDX=\;\frac{\sum_{i=1}^{n}e_{i}}{V_{molecule}}r_{e}$ where *n* is the number of atoms of type i and *e* is the number of electrons. $r_{e}$ is the radius of the electron, 2.8 × 10^−13^ cm. 

The calculated parameters for the relevant components using the available chemical information are summarized in Table 3.

The contrast factors between the significant components were then estimated on the basis of the calculated SLD and are reported in Table 4. Sample E, considered as an ideal two-component system was used to verify the estimated contrast factors with the experimental scattering intensities. Whereas the SLD of SBR and the corresponding contrast of SBR-to-oil in sample E is in good agreement for the SANS case, a clear discrepancy was observed in SAXS, which led to higher-than-expected intensities. Doubts about the absolute calibrated intensity levels can be excluded, however, in view of the elaborate cross-references at different large-scale facilities. The reason for the reversed intensity order is then to be sought in the assumptions about the components. 

Deviations can also be partly due to uncertainties in the thickness of each sample, the statistical and systematic errors of the data themselves, and the quality and intrinsic accuracy of the absolute calibrations. Inaccurate estimation of the SLD of SBR could be also due to the neglect of the components present in phr amounts <2.5 phr. On the other hand, the SLD of the oil in both neutron and X-ray data, calculated from the element analysis can be accurately determined. 

To comply with the aforementioned higher SAXS intensity, the SLD of the SBR in the X-ray case should then be 8.46× 10^10^ cm^−4^, i.e., lower by 4%–5% lower than the theoretical value. It is worth noting that the SANS method is insensitive, in terms of contrast, to the alkane-like compounds of the matrix. The coherent SLD contribution of a CH_2_ section is about 0 for SANS (b_C_ = 6.65 fm, b_H_ = −3.74 fm). This is not the case for SAXS where each atom always adds positively to the electron density. Thus, the optimized value for SBR indicated in Table 4 is assumed to affect only SAXS intensities. The silane component, used as a coupling agent in the filled samples, is in the same way involved in the uncertainty of the SLD determination due to its reaction with both the silica and the polymer matrix. In our estimation, we assume that it is distributed evenly in the matrix.

### 3.3. The Filled Case

The previous relations and considerations applied to the case of unfilled silica rubbers. In the more interesting case of silica-filled systems, the former three-component system now evolves to at least a four-component mixture. A further complication, besides the addition of silica, in the decomposition of the intensities of samples B and C (30 and 60 phr silica, respectively) stems from the presence of the silane component used as a coupling agent. For this reason, the estimation of the contrast factors is based on the estimation of an effective SLD of the matrix including the silane weighted volume fraction. We then obtain again workable equations like before. As in the case of the non-filled rubbers, we neglected all other ingredients with volume fraction below 2.5 phr as they contribute mainly to the incoherent background. The scattering intensity for samples B and C can then be expressed in terms of the partial contributions as:

(6) $$I\left(q\right)={\left(\rho_{sil}-\rho_{eff}\right)}^{2}S_{sil}+{\left(\rho_{ZnO}-\rho_{eff}\right)}^{2}S_{ZnO}+2\left(\rho_{sil}-\rho_{eff}\right)\left(\rho_{ZnO}-\rho_{eff}\right)S_{sil-ZnO}$$

Note the similarity with Sample A, whereas now the effective matrix replaces the oil. Again, the cross term appearing in Equation (6) can be neglected due to the low fraction of ZnO, leading to negligible correlations with silica. The partial form factors can then be expressed using the same approach as before:

(7) $$S_{sil}=\frac{{\left(\rho_{ZnO}-\rho_{eff}\right)}_{X\;}^{2}I_{N}-{\left(\rho_{ZnO}-\rho_{eff}\right)}_{N\;}^{2}I_{X}}{{\left(\rho_{sil}-\rho_{eff}\right)}_{N}^{2}{\left(\rho_{ZnO}-\rho_{eff}\right)}_{X}^{2}-{\left(\rho_{sil}-\rho_{eff}\right)}_{X}^{2}{\left(\rho_{ZnO}-\rho_{eff}\right)}_{N\;}^{2}}$$

(8) $$S_{ZnO}=\frac{{\left(\rho_{sil}-\rho_{eff}\right)}_{N}^{2}I_{X}-{\left(\rho_{sil}-\rho_{eff}\right)}_{X}^{2}I_{N}}{{\left(\rho_{sil}-\rho_{eff}\right)}_{N}^{2}{\left(\rho_{ZnO}-\rho_{eff}\right)}_{X}^{2}-{\left(\rho_{sil}-\rho_{eff}\right)}_{X}^{2}{\left(\rho_{ZnO}-\rho_{eff}\right)}_{N\;}^{2}}$$

where $\rho_{sil}\;$ and $\rho_{eff}\;$ are respectively the SLD for silica and effective matrix including the silane component. In this way, the relevant structure factors of both contributing components can be extracted. A similar approach is lacking up-to-now in the literature. For the first time, microscopic details of the mixing process and/or vulcanization process in a reactive mixture could be accessible.

While interparticle interactions can be excluded for ZnO as well as interactions between the ZnO activator and silica filler particles, the same assumption cannot necessarily be applied to the silica particles. $S_{sil}$ as the cluster form factor generally consists of the product of a particle form factor with an intra-cluster and even an inter-cluster structure factor taking into consideration the interaction between them as we recently showed [31]. However, the full description of the hierarchical structure of the fillers is out of the scope of this work. Here, the decomposition of the different contributions in a mixed-filler system and the evaluation of the ZnO effect is in the foreground. 

In order to obtain a common *q-* range and identical step size between the data points for X-ray and neutron results and allow the weighed subtractions in Equations (7) and (8), a cubic spline is applied to the SAXS data. 

## 4. Results and Discussion

**Figure 1:**
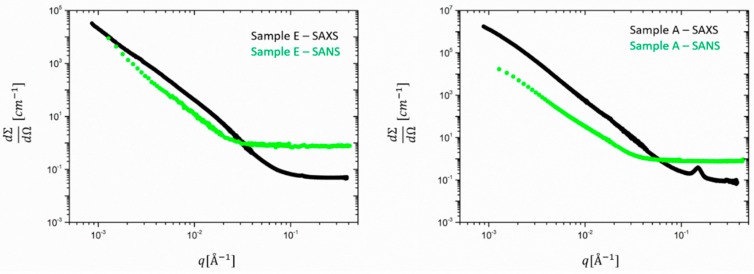
Absolutely calibrated SAXS (black) and SANS (green) data for samples E (left) and A (right). The data are not background-corrected.

First, the unfilled samples are analyzed. Figure 1 shows the comparison between absolutely calibrated total SANS and SAXS results obtained for samples E and A, before the subtraction of the incoherent background. For sample A, the contribution of 2.5 phr ZnO is immediately evidenced by a ~ 10-fold increased intensity between A and E and an associated different *q*-dependence in the q-range 10^−3^ < q < 10^−2^ A^−1^ and a crystalline Bragg peak around 0.15 A^−1^.

**Figure 2:**
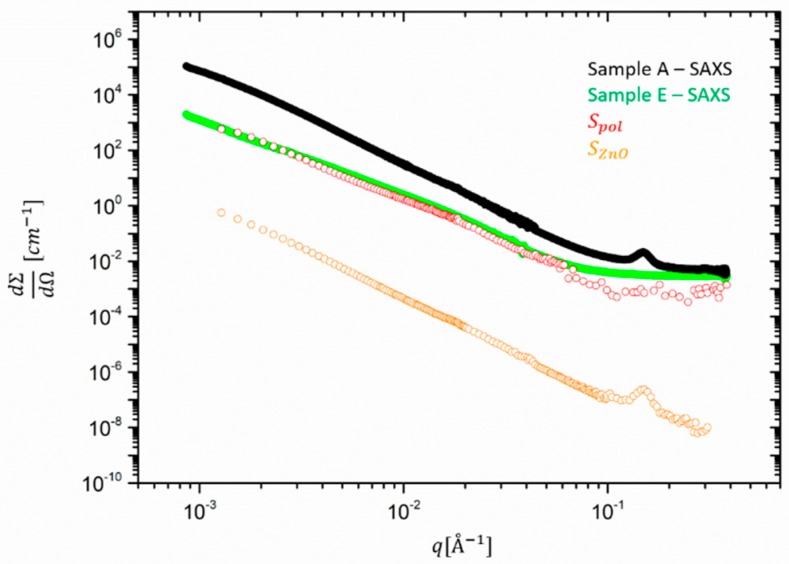
Partial structure factors $S_{pol}$ (red) and $S_{ZnO}$ (orange) as derived from sample A in comparison with absolutely calibrated SAXS data for samples A (black) and E (green). $S_{pol}$ was arbitrarily shifted to be compared with sample E.

In Figure 2, we compare extracted *S_po_*_l_ and *S_Zn0_* with the SAXS intensities of sample A and E. The contribution of the ZnO form factor to the SANS curves results to be irrelevant as we show later. The partial form factors $S_{pol}$ and $S_{ZnO}$, respectively in red and orange, were shifted by an arbitrary factor along the *y* axis for the sake of comparison with the experimental data.

The figure shows that the scaled $S_{pol}$ as derived from sample A is perfectly congruent with the intensity of sample E, leading to the conclusion that—maybe unexpectedly—the vulcanization with and without ZnO would not lead to differences in the matrix structure detectable by scattering in length scales smaller than ~3000 Å. On the other hand, $S_{ZnO}$ shows a visible power law contribution for *q <* 10^−1^*,* indicative for additional scattering to $S_{pol}$.

The same approach defined for the extraction of the single contributions from the total scattering function can be transferred to the case of the filled samples.

**Figure 3:**
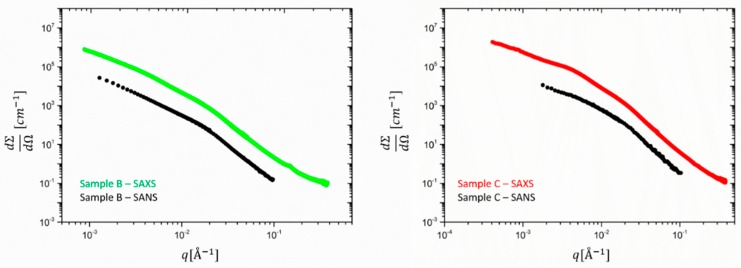
Experimental SANS and SAXS data in absolute intensity units cm^−1^ for the ZnO-containing filled rubbers, sample B (left) and sample C(right). The number of data points of the ultrahigh-resolution SAXS data was about 4500 and was reduced to a SANS-compatible lower resolution of about 350 data points by means of a cubic spline fitting procedure.

Going to filled rubbers, Figure 3 shows a comparison between the SAXS and SANS averaged scattering intensities obtained for samples B and C (respectively, 30 and 60 phr silica). Besides the different absolute intensity and *q*-range due to instrumental factors, SANS and SAXS curves show different low-to-intermediate *q* < 0.01 Å^−1^ behavior. In addition, it can be seen that for *q* > 0.01 Å^−1^ the silica particle structure dominates in both SAXS and SANS. 

The power law exponent in this scale range differs from −4 and stands for a deviation from the ideal spherical particle behavior. The value of approximately −3.7 observed here was also found in the literature for the same type of silica particles. Since this slope is not affected by particle polydispersity it indicates a rough silica surface. For the SANS data of sample C a closer inspection of the lower *q* < 0.01 Å^−1^ reveals a power law region which is the signature of silica aggregates with a mass fractal dimension of ~2.2 and an onset of a Guinier plateau is seen. In sample B a Guinier region cannot be detected due to the obvious formation of larger clusters. This finding corroborates the finding that the cluster size decreases when the filling degree is increased. In addition, some orientation seems to be visible in the higher filling degree sample (C), and therefore, the data must be analyzed along two perpendicular directions. We have selected only the vertical direction as it allows obtaining lower *q*-values due to the horizontal alignment of the beam stop stick in SAXS. 

**Figure 4:**
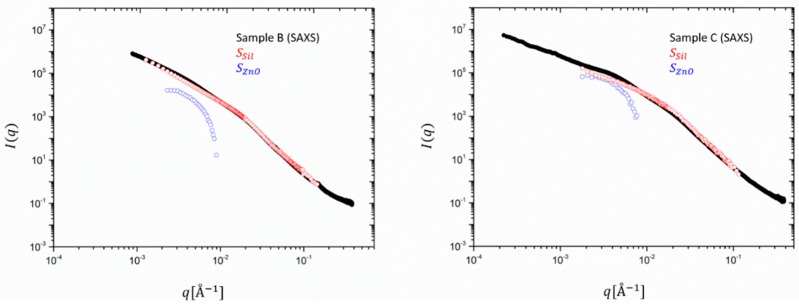
Experimental SAXS data in absolute intensity units cm^−1^ for samples B (30 phr) (left) and C (60 phr) (right). Partial form factors for silica ($S_{pol}$, red) and ZnO ($S_{ZnO}$, blue) were multiplied by the contrast factors to re-construct the intensity.

Besides the different extension in the *q*-range due to instrumental factors, the SAXS data show in both cases a shallow peak or shoulder around *q* ~ 4 10^−^^3^ Å^−1^, which cannot be seen in the SANS results. This is attributed to ZnO, which has a much stronger contribution in the X-ray intensity than in the neutron data, as already observed for the unfilled rubbers. Interestingly, sample C presents a more pronounced peak with respect to sample B, probably due to the lower contribution of the smaller clusters to the scattering function. In Figure 4, the partial contributions $S_{ZnO}\;$ and $S_{Sil}\;$ are multiplied by the contrast factors, as shown in Equation (6) and the experimental SAXS intensities are re-constructed. ZnO contributes to 10% to the total intensity for sample B (30 phr) but amounts to ~50% for C. The scattering at high q is fully determined by silica and consolidates a rather particle-like morphology for ZnO, its form factor dropping with *q^−4^*.

In addition and as anticipated, the power law exponent of approximately −2, found in the ultra-low *q* region of the SAXS data could be attributed to the connectivity of clusters into a network when the volume fraction is increased above their percolation threshold [2,3,4]. 

**Figure 5:**
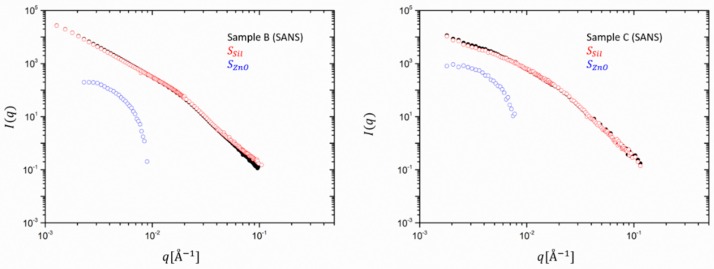
Experimental SANS data in absolute intensity units cm^−1^ for samples B (30 phr) (left) and C (60 phr) (right). Partial form factors for silica ($S_{pol}$, red) and ZnO ($S_{ZnO}$, blue).

Except for these observations, however, the main result and highlight of this first decomposition of SAXS intensities in partial structure factors is the important extraction of the scattering contribution of minute amounts of ZnO and some microscopic details from a clear *q*-dependence. A comparison with Figure 5 showing the same decomposition for SANS data, highlights that due the different contrast, the contribution of ZnO to the total scattering intensity is about two orders of magnitude lower than the one of the silica. However, even the catalytic content of ZnO impedes the analysis in the full *q* range since the intensity of the extracted partial structure factor drops very fast to below the sensitivity limit of the SAXS or SANS machine. Both SANS curves highlight that the main contribution comes almost entirely from silica. The catalyst has an almost negligible effect on the scattering profile. In SAXS, however, both partial intensities contribute to almost the same amount.

Thus, this analysis allowed us to evaluate the total scattering intensities as the sum of contributions of 2 components, similar to the case of a dual filler system. The structure of both in the mixture, as well as their mutual influence, can thereby be captured. 

Let’s first discuss ZnO. The stronger effect of the ZnO contribution in sample C over B is tentatively correlated to a simultaneous decrease of the silica contribution of smaller clusters. 

**Figure 6:**
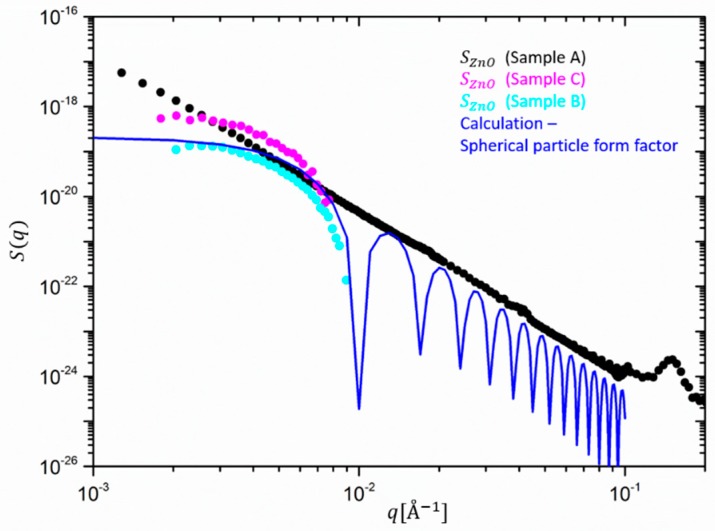
Partial structure factors for ZnO extracted from sample A (black), B (cyan), and C (magenta), and theoretical calculation of a spherical particle form factor (blue line).

The structure of ZnO, i.e., their *q*-dependence in the filled rubbers is, however, very different in comparison to that, extracted from the unfilled sample, as shown in Figure 6. In the case of sample A, the full scattering range could be accessed, and a typical power-law-like behavior as for silica is found. The slope at high *q* is close to −3.8 and indicates some deviation from smooth spherical behavior. Interestingly, the ZnO partial structure factor extracted from sample A does not exhibit any Guinier-like behavior at the lowest *q*, as shown by samples B and C. This indicates that either large ZnO clusters are formed in the case of the unfilled rubber in comparison to the silica-filled ones, which prevent the appearance of the Guinier region in the accessible *q*-range, or huge micron-sized strongly polydisperse and rough particles are still present, the size of which is larger than *R* > 1/q_min_ =1000 Å =0.1 μ.

The situation is entirely different if ZnO is mixed into the rubber together with a much larger amount of silica. Figure 6 clearly shows that after the mixing and vulcanization rather monodisperse single ZnO particles are dispersed, although the high *q* region cannot be detected precisely anymore. We confirm this by comparing with a calculation of a monodisperse particle form factor (blue line in Figure 6). The computed intensity of a single ZnO particle follows in reasonable agreement the calculated spherical form factor, i.e., the Guinier plateau and the characteristic steep drop of the order of about 100 at q ~ 0.01 Å^−1^, i.e., *qR* ~ *4.5*. The silica nanofillers, therefore, seem to affect the size of the ZnO nanocrystals and/or their aggregation. This could be explained considering the different shear forces imposed on the sample during the milling process. Such forces typically increase due to the presence of silica fillers in the rubber, which increase the melt viscosity by orders of magnitude and lead to a break-down of ZnO aggregates or crystals.

**Figure 7:**
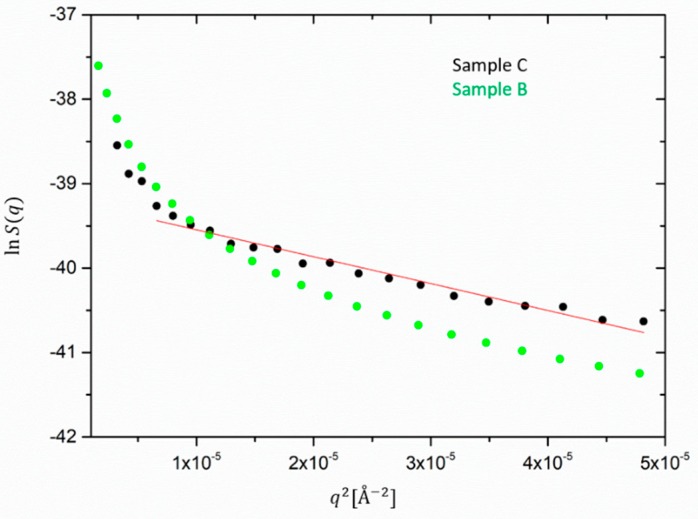
Guinier extrapolation for the silica cluster radius for sample C (black). The fit cannot be uniquely applied to sample B (green) due to the strong deviation from linearity.

**Figure 8:**
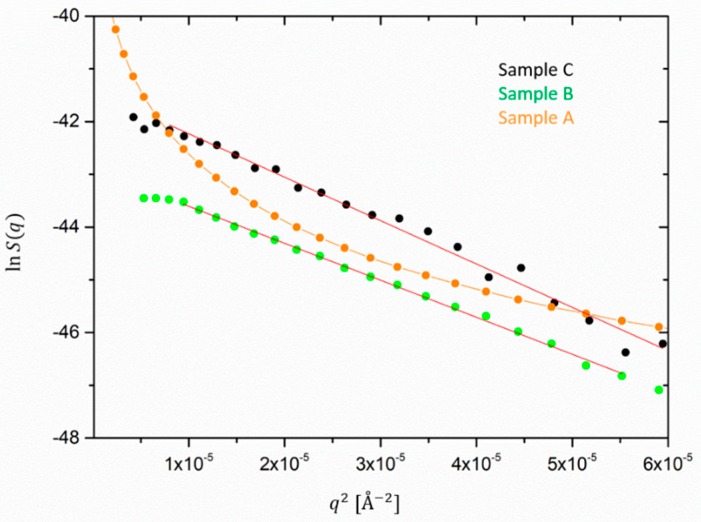
Guinier extrapolation of the ZnO radius for sample C (black) and B (green). Sample A (orange) clearly shows a deviation from the Guinier dependence.

In order to compare the size of silica aggregates and ZnO species in the filled samples, the low *q* scattering for both silica and ZnO partial structure factors is presented in a Guinier-type representation in Figure 7 and Figure 8. The slopes are then related to $–R_{g}^{2}/3.$

Next, silica is discussed. For the silica-extracted form factor, shown in Figure 7, an average radius of gyration *R_g_* can be determined only for sample C because sample B does not exhibit a Guinier-like behavior or is more polydisperse. A cluster size of about 280 Å is obtained from the linear fit of the Guinier plot of $S_{sil}\left(q\right)$ and an increase is expected with a lower volume fraction. However, it has to be taken into account that the extraction of the silica cluster size from the Guinier-like description consists also of an approximation, as sample C does show a deviation from the simple Guinier behavior at low *q.* This extrapolation neither takes into account the polydispersity nor the interactions between the clusters. A more precise estimation of the silica cluster sizes is out of the scope of this work. A fit to a hierarchical scattering model is treated in a recently published work [31].

For samples B and C the extrapolation of the ZnO size, reported in Figure 8 yields almost comparable sizes of about 450–480 Å. It could not be applied to the ZnO form factor extracted from sample A as its low *q*-dependence clearly deviates from the Guinier behavior. Note that the maximum detectable size in the available q-range would approximately be ~1000 Å. Thus, the size of ZnO does not differ considerably from the silica aggregates in the case of the filled samples. Therefore, we infer that in the presence of silica, the processing conditions probably have a comparable effect on the de-agglomeration or destruction of the ZnO granulates. Besides this, the values found for the ZnO radius are comparable with the ones reported in the literature [22]. In other works, the size determination by means of other techniques for ZnO dispersed particles ranged from 300 to 500 Å as the result of the balance of fracture of aggregates and material resistance, i.e., intrinsic hardness in milling processes [32,33].

## 5. Conclusions

We have shown that in industrially mixed rubber compounds a decomposition of the SAXS scattering intensity into its single contributing components, i.e., silica and ZnO is possible. We showed that SANS is hardly affected as it was postulated. The structure factor, the size and morphology of the silica aggregates and of the ZnO nanocrystals as an additional element of analysis can now be obtained. Latter strongly depends on the presence of silica during the mixing process. We observe that ZnO in silica-free samples forms aggregates or is of the micron-size and is characterized by a fractal surface. It appears to be structured on the same length scale as the silica filler. Therefore, a dependence of the size on the shear force exerted on the samples during the mixing process is suspected. Furthermore, the size of the ZnO catalyst is nearly constant for filled samples containing 30 and 60 phr of silica. As a consequence of this study, corrections for ZnO scattering based on computation with well-estimated contrast factors could be formulated. With estimates for the size of the resulting particle and assumptions for the average SLD of the effective matrix for the contrast, the contribution of $S_{ZnO}$ can be calculated theoretically for standard SAXS analyses. The proposed analysis is general and can be applied in the future to novel dual filler systems, which scatter both but differently strong in both SANS and SAXS.

## Figures and Tables

**Table 1:** Composition of investigated samples. From left to right column the complexity is decreased. The compositions are given in parts per 100 g rubber (phr).

Component	phr			
	Sample C (60 phr)	Sample B (30 phr)	Sample A (unfilled)	Sample E (unfilled, no ZnO)
SBR	137.5	137.5	137.5	137.5
Antioxidant	0.75	0.75	0.75	0.75
Oil	2	2	2	2
Activator	3	3	3	-
Silica 1165MP	60	30	-	-
Silane Si266	4.8	2.4	-	-
Antioxidant	1.75	1.75	1.75	1.75
Antioxidant	0.5	0.5	0.5	0.5
ZnO	2.5	2.5	2.5	-
Sulfur	0.8	0.8	0.8	0.8
Accelerator	2.4	2.4	2.4	2.4
Accelerator	1.5	1.5	1.5	1.5

**Table 2:** Silica content in the different samples. phr is parts per 100 g of rubber.

Sample	Total phr	Spec Gravity	Silica phr	Silica Density $\left(g/cm^{3}\right)$	Silica Volume Fraction (ϕ)
C	217.5	1.14	60	2.2	0.143
B	185.1	1.07	30	2.2	0.079
A	152.7	0.98	0	2.2	0
E	147.2	0.97	0	2.2	0

**Table 3:** Mass densities and scattering length densities for the most significant components of the mixture (amount > 2.5 phr). Only for SBR, the experimental value for the SLD in the case of X-rays was optimized against experimental data (see text).

Component	Mass density g/cm^3^	SLD neutron cm^−2^	SLD X-ray cm^−2^	Remarks
Oil	0.94	1.036 × 10^10^	8.70 × 10^10^	C_7.4_H_8.4_
SBR	0.95	0.910 × 10^10^	8.46 × 10^10^ opt.	C_5.6_H_6.8_ 40% styrene
Silica	2.10	3.320 × 10^10^	17.9 × 10^10^	Solvay 165 m^2^/g
ZnO	5.60	4.760 × 10^10^	43.3 × 10^10^	BET 5m^2^/g
Coupling agent Silane (Si 266)	1.03	0.149 × 10^10^	9.54 × 10^10^	C_18_H_42_O_6_S_2_Si_2_
**Filled rubber samples**	**0.95**			**SBR/oil/silane:** *v*/*v***fractions**
Effective Matrix (B) Effective Matrix (C)		0.929 × 10^10^ 0.905 × 10^10^	8.546 × 10^10^ 8.576 × 10^10^	0.71/0.27/0.02 0.69/0.26/0.05.

**Table 4:** Contrasts for SANS and SAXS analysis.

**Neutron Contrasts [cm^−4^]**	**Oil**	**SBR**	**Silica**	**ZnO**
**Oil**	-			
**SBR**	1.58 × 10^18^	-		
**Silica**	5.22 × 10^20^	5.81 × 10^20^	-	
**ZnO**	13.87 × 10^20^	14.82 × 10^20^	2.07 × 10^20^	-
**Filled rubber samples**	-	-	B: 5.72 × 10^20^ C: 5.83 × 10^20^	B: 14.67 × 10^20^ C: 14.86 × 10^20^
**X-ray** **contrasts [cm^−4^]**	**Oil**	**SBR**	**Silica**	**ZnO**
**Oil**				
**SBR**	5.76 × 10^18^	-		
**Silica**	84.64 × 10^20^	89.1 × 10^20^	-	
**ZnO**	119.7 × 10^21^	121.4 × 10^21^	6.45 × 10^22^	-
**Filled rubber samples**	-	-	B: 87.50 × 10^20^ C: 86.94 × 10^20^	B: 120.8 × 10^21^ C: 120.6 × 10^21^
